# Supplementary material for: Facilitators and barriers to uptake of digital adherence technologies in improving TB care in Ethiopia: A qualitative study
Source: PLOS Digit Health. 2024 Nov 21;3(11):e0000667. doi: 10.1371/journal.pdig.0000667 (PMC11581308; doi:10.1371/journal.pdig.0000667)
Supplement: S1 Appendix — (DOCX) [file pdig.0000667.s001.docx]

**Annex 1: Characteristics of participants**

| 1. **People with drug sensitive TB** | | | | | |
| --- | --- | --- | --- | --- | --- |
| **S.no** | **Participant ID** | **Age (years)** | **Gender** | **DAT type** | **Region** |
| 1 | PWTB #1 | >55 | M | Smart pillbox | Addis Ababa |
| 2 | PWTB #2 | 46-55 | F | Label | Addis Ababa |
| 3 | PWTB #3 | 36-45 | M | Label | Addis Ababa |
| 4 | PWTB #4 | 26-35 | M | Label | Addis Ababa |
| 5 | PWTB #5 | >55 | M | Label | Addis Ababa |
| 6 | PWTB #6 | 18-25 | M | Smart pillbox | Addis Ababa |
| 7 | PWTB #7 | 36-45 | F | Smart pillbox | Addis Ababa |
| 8 | PWTB #8 | 18-25 | M | Smart pillbox | Addis Ababa |
| 9 | PWTB #9 | 46-55 | M | Smart pillbox | Addis Ababa |
| 10 | PWTB #10 | 26-35 | M | Smart pillbox | Addis Ababa |
| 11 | PWTB #11 | 26-35 | F | Smart pillbox | Addis Ababa |
| 12 | PWTB #12 | >55 | M | Label | Addis Ababa |
| 13 | PWTB #13 | 46-55 | F | Label | Addis Ababa |
| 14 | PWTB #14 | 26-35 | F | Label | Addis Ababa |
| 15 | PWTB #15 | 26-35 | F | Label | Addis Ababa |
| 16 | PWTB #16 | 18-25 | F | Smart pillbox | Oromia |
| 17 | PWTB #17 | 18-25 | M | Smart pillbox | Oromia |
| 18 | PWTB #18 | 26-35 | F | Smart pillbox | Oromia |
| 19 | PWTB #19 | 18-25 | F | Label | Oromia |
| 20 | PWTB #20 | 18-25 | F | Label | Oromia |
| 21 | PWTB #21 | 18-25 | M | Label | Oromia |
| 22 | PWTB #22 | 18-25 | F | Label | Oromia |
| 23 | PWTB #23 | 18-25 | F | Smart pillbox | Oromia |
| 24 | PWTB #24 | 26-35 | M | Smart pillbox | Oromia |
| 25 | PWTB #25 | 46-55 | M | Label | Addis Ababa |
| 1. **People with multi-drug resistant TB** | | | | | |
| 26 | PWTB #26 | 26-35 | M | Smart pillbox | Addis Ababa |
| 27 | PWTB #27 | 26-35 | M | Smart pillbox | Addis Ababa |
| 28 | PWTB #28 | 26-35 | F | Smart pillbox | Addis Ababa |
| 29 | PWTB #29 | 26-35 | F | Smart pillbox | Addis Ababa |
| 30 | PWTB #30 | 26-35 | F | Smart pillbox | Addis Ababa |
| 31 | PWTB #31 | 26-35 | M | Smart pillbox | Addis Ababa |
| 32 | PWTB #32 | 26-35 | F | Smart pillbox | Addis Ababa |
| 33 | PWTB #33 | >55 | M | Smart pillbox | Addis Ababa |
| 1. **Focal healthcare workers** | | | | | |
| 1 | HCW #1 | <30 | F | Label | Addis Ababa |
| 2 | HCW #2 | 40-49 | M | Smart pillbox | Addis Ababa |
| 3 | HCW #3 | 30-39 | F | Label | Addis Ababa |
| 4 | HCW #4 | 30-39 | F | Label | Addis Ababa |
| 5 | HCW #5 | 40-49 | M | Smart pillbox | Addis Ababa |
| 6 | HCW #6 | 30-39 | F | Smart pillbox | Addis Ababa |
| 7 | HCW #7 | 30-39 | F | Smart pillbox | Addis Ababa |
| 8 | HCW #8 | <30 | F | Smart pillbox | Addis Ababa |
| 9 | HCW #9 | 40-49 | F | Label | Addis Ababa |
| 10 | HCW #10 | <30 | F | Label | Addis Ababa |
| 11 | HCW #11 | 30-39 | F | Smart pillbox | Oromia |
| 12 | HCW #12 | <30 | M | Label | Oromia |
| 13 | HCW #13 | >50 | F | Label | Oromia |
| 14 | HCW #14 | 30-39 | M | Smart pillbox | Oromia |
| 15 | HCW #15 | 30-39 | F | Smart pillbox | Oromia |
| 16 | HCW #16 | >50 | M | Label | Oromia |
| 17 | HCW #17 | 40-49 | F | Label | Oromia |
| 18 | HCW #18 | >50 | F | Smart pillbox | Oromia |
| 19 | HCW #19 | 30-39 | F | Label | Oromia |
| 20 | HCW #20 | 30-39 | F | Smart pillbox | Oromia |
| **4. Stakeholders in TB care** | | | | | |
| 1 | STK #1 | na | M | Na | national representative |
| 2 | STK #2 | na | M | Na | national representative |
| 3 | STK #3 | na | M | Na | national representative |
| 4 | STK #4 | na | M | Na | national representative |

DAT digital adherence technology; PWTB person with TB; HCW health care worker; M male; F female; Na not applicable

| **5. Number of health facilities per participant group** | | | | | | | |
| --- | --- | --- | --- | --- | --- | --- | --- |
| Region | Pillbox | | Labels | | Total | | People with MDR-TB (all facilities implemented smart pillboxes) |
|  | PWTB | HCWs | PWTB | HCWs | PWTB | HCWs |  |
| Addis Ababa | 3 | 5 | 4 | 5 | 7 | 10 | 6 |
| Oromia | 3 | 5 | 2 | 5 | 5 | 10 |  |
| Total | 6 | 10 | 6 | 10 | 12 | 20 |  |

PWTB person with TB; HCW health care worker; MDR multi-drug resistant TB
