# Supplementary material for: Facilitators and barriers to uptake of digital adherence technologies in improving TB care in Ethiopia: A qualitative study
Source: PLOS Digit Health. 2024 Nov 21;3(11):e0000667. doi: 10.1371/journal.pdig.0000667 (PMC11581308; doi:10.1371/journal.pdig.0000667)
Supplement: S2 Appendix — (DOCX) [file pdig.0000667.s002.docx]

**Annex 2: Interview Guides**

**2.1. Patients’ interview guide – smart pill box users**

| Areas of Inquiry | Main Topics | Possible Probes |
| --- | --- | --- |
| Experience, attitude, feeling and level of satisfaction of the differentiated model of care and use of the <smart pill box> (motivators and barriers) | *Who explained to you on how to use the <smart pill box> and how did you feel about this?* | - *How long was the explanation?* - *Was it easy to understand how to use the smart pill box based on the explanation?* - *Is there anything that you would change about how you were informed about using the smart pill box?* |
|  | *How easy was it for you to use the <smart pill box>?* | - *What aspect of the smart pill box was easy to use?* |
|  | *Please describe any difficulties you experienced when using the <smart pill box>.* | - *Was you able to send SMSs daily?* - *If not, what where the problems (no phone, power issues, problems sending a text - relative helping with sending texts)* |
|  | *What were your concerns about using or having the <smart pill box>?* | - *Did anyone ask about the smart pill box? (did you tell anyone ?). if yes, was it difficult to explain to someone about this?* - *Did anyone notice when you open your pill box and asked you about it?* |
|  | *How did you feel about telling people about the <smart pill box>?* | - *Do you found it easy to tell people about TB treatment using smart pill box?* - *Who did you tell and why? (if any). If yes, how was their reaction?* |
|  | *Have you ever experienced accidental or multiple opening of box/day?* | - *If yes, what was the cause?* - *When was this happened since you started to use it?* - *Where was you keep/store it?* |
|  | *What was helpful about using the <smart pill box> for TB treatment?* | - *For example: the reminder function, adequate information from HCWs or other sources, etc* |
|  | *What was difficult about using the <smart pill box> for TB treatment?* | - *What challenges have you faced while using the smart pill box?* |
|  | *Have you experienced differentiated model of care? If yes, how was your overall experience?* | - *Have you received automated SMS reminders when you forgot to take your treatment or box not opened? Can you describe your experience?* - *Can you describe your experience of being part of the follow-up phone calls?* - *Can you describe your experience of being part of the home visits?* |
|  | *Please describe any cultural or traditional barriers to the use of the <smart pill box> and differentiated model of care* | - For example:- difficulty to tell people that you are using the <smart pill box>, negative perceptions around DATs, issues related to disclosure of TB status e.t.c - Difficulties related to home visit by HCWs (e.g., what was the reaction from other (neighbours or family members)? |
|  | *Please describe your level of satisfaction with use of the <smart pill box>* | - Please describe how the small pill box helped you not to miss taking of your medication? - Please describe how the use of smart pill box reduced number of appointment for refill - Overall, how do you describe your satisfaction about using the smart pill box? |
|  | *What would make it easier for you to use the <smart pill box>?* | - *For example: volume of reminder beeps, voice reminders instead of sms, connectivity issues, language preference etc)* |
| Perceptions, experiences and feelings toward to the retention activities [only ask if relevant] | *How did you feel about receiving reminder SMSs?* | - *What did you like or dislike about this?* |
|  | *How did you feel about receiving phone calls?* | - *What did you like or dislike about this?* |
|  | *How did you feel about staff visiting your home?* | - What did you like or dislike about this? |
|  | *Please describe your experience of the counselling received?* | - What did you like or dislike about this? |
|  | *Of all the activities that you were part of during this study, which was most influential in helping you take your TB treatment and which did you find was not useful? Please elaborate* | - Thinking about the differentiated care model ( sms, phone call and home visits) which was most relevant to take you TB treatment and which was not much useful? |
|  | *Can you describe any gaps which exist in the way the intervention was delivered currently?* | - What can be improved? |
| `wrap-up | *Are there any final thoughts you have about the differentiated model of care and use of the <smart pill box>?* | - Do you have any final comment related to the use of smart pill box? - Do you have any final comment related to the being part of the differentiated care? |
|  | Now we have come to the end of our discussion. Thank you for your participation. If you have any questions about your study participation, please contact us. Thank you. | - Refer them to the PIS for whom and how they can contact for any further information about the study |

**2.2. Patients’ interview guide – label users**

| Areas of Inquiry | Main Topics | Possible Probes |
| --- | --- | --- |
| Experience, attitude, feeling and level of satisfaction of the differentiated model of care and use of the <label> (motivators and barriers) | *Who explained to you on how to use the <label> and how did you feel about this?* | - *How long was the explanation?* - *Was it easy to understand how to use the label based on the explanation?* - *Is there anything that you would change about how you were informed about using the label?* |
|  | *How easy was it for you to use the <label>?* | - *What aspect of the label was easy to use?* |
|  | *Please describe any difficulties you experienced when using the <label>.* | - *Was you able to send SMSs daily?* - *If not, what where the problems (no phone, power issues, problems sending a text - relative helping with sending texts)* |
|  | *What were your concerns about using the <label>?* | - *Did anyone ask about the label? (did you tell anyone ?). if yes, was it difficult to explain to someone about this?* - *Did anyone notice when you send the codes through sms and asked you about it?* |
|  | *How did you feel about telling people about the <label>?* | - *Do you found it easy to tell people about TB treatment using label method?* - *Who did you tell and why? (if any). If yes, how was their reaction?* |
|  | *What was helpful about using the <label> for TB treatment?* | - *For example: adequate information from HCWs or other sources, etc* |
|  | *What was difficult about using the <label> for TB treatment?* | - *What challenges have you faced while using the label?* |
|  | *Have you experienced differentiated model of care? If yes, how was your overall experience?* | - *Have you received automated SMS reminders when you forgot to take your treatment or box not opened? Can you describe your experience?* - *Can you describe your experience of being part of the follow-up phone calls?* - *Can you describe your experience of being part of the home visits?* |
|  | *Please describe any cultural or traditional barriers to the use of the <label> and differentiated model of care* | - For example:- difficulty to tell people that you are using the <label>, negative perceptions around DATs, issues related to disclosure of TB status e.t.c - Difficulties related to home visit by HCWs (e.g., what was the reaction from other (neighbours or family members)? |
|  | *Please describe your level of satisfaction with use of the <label>* | - Please describe how the label helped you not to miss taking of your medication? - Please describe how the use of label reduced number of appointment for refill - Overall, how do you describe your satisfaction about using the label? |
|  | *What would make it easier for you to use the <label>?* | - *For example: voice reminders instead of sms, connectivity issues, language preference etc* |
| Perceptions, experiences and feelings toward to the retention activities [only ask if relevant] | *How did you feel about receiving reminder SMSs?* | - *What did you like or dislike about this?* |
|  | *How did you feel about receiving phone calls?* | - *What did you like or dislike about this?* |
|  | *How did you feel about staff visiting your home?* | - What did you like or dislike about this? |
|  | *Please describe your experience of the counselling received?* | - What did you like or dislike about this? |
|  | *Of all the activities that you were part of during this study, which was most influential in helping you take your TB treatment and which did you find was not useful? Please elaborate* | - Thinking about the differentiated care model ( sms, phone call and home visits) which was most relevant to take you TB treatment and which was not much useful? |
|  | *Can you describe any gaps which exist in the way the intervention was delivered currently?* | - What can be improved? |
| `wrap-up | *Are there any final thoughts you have about the differentiated model of care and use of the <label>?* | - Do you have any final comment related to the use of label? - Do you have any final comment related to the being part of the differentiated care? |
|  | Now we have come to the end of our discussion. Thank you for your participation. If you have any questions about your study participation, please contact us. Thank you. | - Refer them to the PIS for whom and how they can contact for any further information about the study |

**2.3. HCWs’ Interview Guide**

| Areas of Inquiry | | Main Topics | | Probes | |
| --- | --- | --- | --- | --- | --- |
| 1. Introduction | | What is the title of your current position?    When it comes to patient care and counselling, what are your role and responsibilities?’ | | - *How long have you held this position?* - *Probes for roles and responsibilities in TB Care: diagnostics, counselling, consenting, monitoring of adherence, communication upon the missed doses, clinical examination, home visits* | |
|  |  | How are TB services delivered at your level with regard to the intervention? (i.e. District, Provincial, National) | |  | |
|  |  | In your everyday professional life what do you call the DAT?    How do you call the platform for monitoring of adherence?    How do you call the differentiated model of care? | | - *SmartPill Boxes* - *Medication sleeves* - *Patient Task List* - *EverwellHub* - *Reminder SMS* - *Phone calls to the patients* - *Home visits* - *Counselling* | |
|  |  | (If the interviewee struggles to answer three questions above, they can be reformulated in this way)  If you have to explain what is DAT intervention (including medication device and monitoring platform) to another HCW, who knows nothing about it, what would you tell her/him? | |  | |
|  |  | Please describe your role with the differentiated model of care intervention | | *Are you responsible for the...*   - *Use of Task List* - *Follow up communication with the patients* - *Phone calls, home visits, counselling.* - *What do you do all the time? Frequently? Rare?* | |
|  |  | Please describe the cadre of staff that were involved with delivering the differentiated model of care? | | *How are the responsibilities/functions being shared between you and other HCWs?* | |
| *2.* Feasibility of implementing the differentiated model of care (motivators and barriers) | | Do you remember your expectations about the DAT intervention before it was implemented?    Did you have ones back then? Which ones? | | - *Easier?* - *Convenient?* - *More efficient?* - *Complex?* | |
|  |  | Did your opinion change after implementing intervention? | | *If yes, how?* | |
|  |  | Can you describe the training and resources that staff received prior to or during delivery of the differentiated model of care?    What was your opinion of the training and resources received? | | - *Do you remember the training activities delivered by the research team before you began to use DAT or during the research implementation?* - *What was your first impression?* - *Did you think it was comprehensive?* - *Useful?* - *Sufficient?* - *Has your opinion been changed after the training sessions?* - *Do you have suggestions to improve training e.g who should train, duration, frequency, who should attend, content?* | |
|  |  | From your perspective as a HCW, can you describe the benefits of the differentiated model of care and use of the medication device technology? | | - *Convenience of DAT and Everwell platform* - *Monitoring* - *Use of Task List* - *Relationship with patients*   *(Think of a patient you have been supporting using DAT)* - *Stigma* | |
|  |  | Can you describe challenges of the differentiated model of care and use of the medication device technology? | | - *Network issues* - *Technical glitches* - *Staff turnover/ rotation* - *Support Actions taking more time that it can be* - *Stigma* - *Lack of cell phones* - *Homeless people and drug users* - *Availability of staff to do home visits after 4 days* | |
|  |  | From your perspective as HCWs can TB treatment be improved using this differentiated model of care and the medication device technology? | | *Do you personally think that DAT impact:*   - *Patients’ adherence?* - *Patient-provider relationship?* - *HCW’s workload* - *Relationship with the patient* - *Improved monitoring* - *Training* - *Human Resources* | |
| 3. System level challenges of delivering the intervention Sustainability | | *Please elaborate on the positive changes of the differentiated model of care and use of the medication device technology.* | | - Positive changes to patients - Positive changes to HCWs - Positive changes around the overall TB treatment adherence |  |
|  | | *How do you think these positive changes could be sustained?* | | - What should be done or improved by all parties to keep the above changes sustained |  |
|  | | What could be improved in order to make these positive changes consistent or sustainable? | | - At the level of patients - At the level of HCWs - at the level of health facility - at the level of National TB program (NTP) |  |
|  | | *Please elaborate on challenges of the differentiated model of care and use of the medication device technology. How do you think the challenges could be addressed?* | | - At system level - At HCWs level - At patients level (e.g., Patients opening the box without taking medication or Patients not answering the phone calls made by HCWs |  |
|  | | *Please describe to us what system level structures need to be improved in order to integrate the differentiated model of care and medication device technology into the existing TB program system.* | | - Staff to prepare the boxes - Helpdesk to solve technical issues with medication devices and Everwell Hub - Increase staff numbers - Continuous training to staffs |  |
|  | | *Can you describe to us what systems are in place that could monitor the differentiated model of care and use of the medication device technology?* | | - Do you monitor or capture the challenges, successes or problems of the use of DATs? - Do you capture somehow the issues, challenges and any indicators regarding how you use medication devices and differentiated care model in everyday practice? - Do you capture somehow the issues, challenges and any other indicators of how you use medication technology and differentiated care model in your everyday practice? - Are you doing it personally? - Do you use any kind of system to capture it? - Is there some common tool/approach to do this in your facility shared by other HCWs?’ |  |

**2.4. Stakeholders’ Interview Guide**

| Areas of Inquiry | | **Main Topics** | **Probes** | |
| --- | --- | --- | --- | --- |
| 1. Introduction | | What is the title of your current position? | - Tell us about your overall engagement in TB treatment programs in Ethiopia | |
|  |  | What do you know about the ASCENT project or the DAT intervention we are currently running? | - What are the sources of your knowledge about the ASCENT project? | |
|  |  | Were you engaged with the Ascent project in some way, directly or indirectly? If yes, how? | For example, they may involve as   - Implementation partner - NTP/MOH key stakeholders - Patients' community representatives or community advisory board (CAB) | |
| 2. Feasibility of implementing the differentiated model of care and DATs in Ethiopia (motivators and barriers) | | What were your expectations about the DAT intervention and differentiated model of care before it was implemented? | For example:   - Easier? - Convenient? - More efficient? - Complex? | |
|  |  | What were your some concerns about implementing the DAT and differentiated model of care before its implementation began, considering the Ethiopian context | For example: with respect to   - Patients acceptance - HCWs and other staff involved in the intervention - Facilities where the DAT and differentiated model of care were implemented | |
|  |  | Did your opinion change after the DAT and differentiated model of care implementation started? | - If yes, how? - what is your opinion about the benefits of the interventions? - What practical challenges have you observing during the interventions? | |
|  |  | How do you think the DAT and differentiated model of care changed the following things? It can be positively or negatively? | Probes:   - The capacity and workload of HCWs and staff involved - The quality of patients treatment - The quality of adherence monitoring - Patients' costs related to TB-treatment (e.g., no frequent travel like the DOT) - Patient-provider relationship - TB related Stigma | |
|  |  | Please elaborate on the positive changes of the differentiated model of care and use of the medication device technology. | - Positive changes to patients - Positive changes to HCWs - Positive changes around the overall TB treatment adherence | |
|  |  | Can you describe challenges of the differentiated model of care and use of the medication device technology? | - Network issues - Technical glitches - Staff turnover/ rotation - Support Actions taking more time that it can be - Stigma - Lack of cell phones /power cut - Homeless people and drug users - Availability of staff to do home visits | |
|  |  | From your perspective as a stakeholder, can TB treatment be improved using this differentiated model of care and the DAT? | Do you personally think that DAT impact:   - Patients’ adherence? - Patient-provider relationship? - HCW’s workload - Improved adherence monitoring - Reduce human Resources | |
| 3. Sustainability and scalability of the DAT and differentiated model of care implementation | How do you think the positive changes we discussed above could be sustained? | | What should be done or improved by all parties to keep the above changes sustained   - At the level of patients - At the level of HCWs - at the level of health facility - at the level of National TB program (NTP) or MOH - At community level |  |
|  | How do you describe the opportunities to expand or scale up the DAT intervention in Ethiopia? | | - What opportunities already exist to expand DATs in TB treatment considering the Ethiopian context |  |
|  | What are the practical requirements for the scale-up of the DATs and differentiated model at a country level? | | - What are the things which need to be improved to scale-up this DAT technology in TB treatment considering the Ethiopian context |  |
|  | Are there specific challenges that could become barriers to scale up the DATs and differentiated model of care in Ethiopia? | | Probes:   - Sufficiency of cadre of staff involved in the implementation - HCWs' acceptability of DATs and motivation to implement it - Sufficiency of training for HCWs and staff involved - Cost of the implementation - Patients and community engagement - Funding - Evidence-based effectiveness - Advocacy sufficiency |  |
| 4. Integration of DAT and Differentiated model of care into the existing TB Care | Please describe to us what need to be improved in order to integrate the differentiated model of care and DAT into the existing TB program system? | | - - Integration with NTP   - Advocacy to health authorities   - Unrestricting age and TB patients profile |  |
|  | What need to be done on the different levels of TB care to ensure the sustainability of the DAT and differentiated model of care once it is integrated into the existing TB program system? | | • At the level of patients  • At the level of HCWs  • at the level of health facility  • at the level of National TB program (NTP) or MOH  • At community level |  |
