# Supplementary material for: Facilitators and barriers to uptake of digital adherence technologies in improving TB care in Ethiopia: A qualitative study
Source: PLOS Digit Health. 2024 Nov 21;3(11):e0000667. doi: 10.1371/journal.pdig.0000667 (PMC11581308; doi:10.1371/journal.pdig.0000667)
Supplement: Attachment — Submitted filename: Review of TB Ethiopia Digital Health.docx [file pdig.0000667.s003.docx]

Review of: Facilitators and barriers to uptake of digital adherence technologies in improving TB treatment adherence in Ethiopia: A qualitative study

The content of the paper is quite interesting, novel and relevant for TB treatment & digital adherence technologies. The main claims of the paper were made clear and a great amount of evidence was included in the paper. Unfortunately, the delivery of the collected data is lacking formality and correct grammar in multiple instances (changes pointed out specifically down below). Along with that the formatting and font/font sizes, headings etc. differ throughout. Besides, the results section would greatly benefit from a more precise display, it is fairly easy to get lost throughout all the different points presented. The discussion would also benefit from implementing future recommendations for practice and research. Finally, it would be interesting to display study protocols, interview guidelines and additional material used during the research period under supporting information. By applying the changes listed down below, this qualitative paper will make for a great paper!

- Title Page, p.1. The font of affiliation #5 does not match with the other font used
- Author summary, p. 3 Font seems to be in grey
- Introduction, p.4, 66-67, wording/phrasing sounds informal, I would take out (excluding COVID-19) as emphasis is put on “first leading infectious killer”, remains true regardless of the appearance of COVID-19
- Study Design & Participants, p.5, 103 Please specifiy who is meant with key stakeholders (e.g. religious/community/ etc. leaders?)
- Study Design & Participants, p.5, 112, Incorrect spelling: take out “A” at the beginning of the sentence
- Interview process, p. 6, 116, incorrect spelling: degrees -> plural
- Interview process, p.6, 116: Has there been adequate training/ assistance concerning the cultural background of the study setting?
- Data Analysis, p.6, Font size differs?
- Data Analysis, p.6-7, elaborate on pseudonymization or anonymization? Elaborate on whether transcripts etc. were securely stored, when transcripts and notes will be deleted etc.
- Ethical considerations, p.7, 153, informal spelling: can’t -> cannot
- Characteristics of participants, p.8, 165, incorrect phrasing: include: The majority OF healthcare workers
- Facilitators of DATs uptake, p.11, 199, What does XX stand for at the end of the sentence?
- Facilitators of DATs uptake, p.13 Formatting is off
- Facilitators of DATs uptake, p. 15, 276, font and font size differs
- Reduce workload and work-related TB exposure to providers, p.16, 294: missing letter: ReduceD, 295, missing word, add “a” few
- Theme 2, p.16, 306, Heading is misspelled, is it supposed to mean Easy for use?
- Theme 3, p.18, 351, Wording: important others? Are they referring to “important significant others”?
- Theme 3, p.18, 355, wording, change to: “in their TB treatment”
- Theme 4, p. 19, 362, missing word: can increase “THE” uptake
- Overall heading stile is not coherent
- Barriers for uptake of DATS, p.20, 390, missing word: those “WHO” did not have phones, 392, missing word: to “THE” implementation, 394, missing word: can result “IN THE” fear of stigma
- Theme “1” Network and electricity issues, p.21, 397, Duplicate Heading, Theme one already exists, maybe it is best to change it to Theme 1.2. ? 404, wrong spelling: my? Do they mean: may?, 428, PWTB is always addressed singularly, needs to be plural, add an “s”, 447, missing word: they “HAD” forgotten.., 448: missing words, may consider them “TO BE” non-adherent, come to “THE” health facility
- Theme 2: Lack of inclusivity, p. 24, 459,missing word: As “A” barrier, 460 odd sentence: those did not have phones?, people in prison: is that not too far-fetched since this target group is not included here?
- Theme 3: Fear of Stigma etc., p.24, 473, missing word: was
- Discussion, p.25, 495-496, phrasing is odd
- Strenghts and limitations, p. 27, 537, missing word “THE” capturing, 538, missing letter: engaging diverse “groupS”, including “DRUG SENSITIVE PEOPLE”, 50 is “THAT” it adds, 547: DATS font is different
