# Supplementary material for: Facilitators and barriers to uptake of digital adherence technologies in improving TB care in Ethiopia: A qualitative study
Source: PLOS Digit Health. 2024 Nov 21;3(11):e0000667. doi: 10.1371/journal.pdig.0000667 (PMC11581308; doi:10.1371/journal.pdig.0000667)
Supplement: Attachment — Submitted filename: Response to Reviewers.docx [file pdig.0000667.s004.docx]

To: Margaret Isioma Ojeahere, MBBS, FWACP
Academic Editor, PLOS Digital Health

24 June 2024

Dear Dr Ojeahere

Re: PDIG-D-23-00479 (Facilitators and barriers to uptake of digital adherence technologies in improving TB treatment adherence in Ethiopia: A qualitative study)

We would like to thank the reviewers for their comments and we are honoured to be given the opportunity to respond to the points they raised. We have outlined our responses to all comments below and indicated where there are major changes in the text.

We have also made small changes to the text throughout to make the manuscript more readable and included Annex 1, a description of the study participants and the added the interview guides in Annex 2.

Finally, we have reviewed the references and have made some changes - in particular, we removed the reference Crowder R et al (BMJ open. 2020;10(11):e039895; error in our referencing) and updated the reference to the WHO Global TB report from 2022 to 2023. Reference #13 is a new addition.

Yours sincerely

Zewdneh Shewamene on behalf of all co-authors

| Reviewer Comments | Response |
| --- | --- |
| Reviewer One |  |
| The content of the paper is quite interesting novel and relevant for TB treatment & digital adherence technologies. The main claims of the paper were made clear and a great amount of evidence was included in the paper. Unfortunately the delivery of the collected data is lacking formality and correct grammar in multiple instances (changes pointed out specifically down below). Along with that the formatting and font/font sizes headings etc. differ throughout. Besides the results section would greatly benefit from a more precise display it is fairly easy to get lost throughout all the different points presented. The discussion would also benefit from implementing future recommendations for practice and research. Finally it would be interesting to display study protocols interview guidelines and additional material used during the research period under supporting information. By applying the changes listed down below this qualitative paper will make for a great paper! | We appreciate the positive feedback on our paper's content and relevance. We have thoroughly reviewed and corrected all grammatical errors and formatting issues.  We reorganized the results section to ensure clarity and ease of understanding.  We have included an “implications” section in the discussion to highlight the relevance of this study to practice and research. We have provided interview guidelines, and other supplementary materials in the supporting information (See Annex 1 and 2 from the supplemental files). The study protocol for the larger study has been published and cited in this paper (See ref #14; P.6, 115). |
| 1. Title Page p.1. The font of affiliation #5 does not match with the other font used | Corrected for the font inconsistency on the title page. |
| 2. Author summary p. 3 Font seems to be in grey | The font colour has been revised in the author summary to match the rest of the document. See P.2 |
| 3. Introduction p.4 66-67 wording/phrasing sounds informal I would take out (excluding COVID-19) as emphasis is put on “first leading infectious killer” remains true regardless of the appearance of COVID-19 | We have revised the phrasing and removed the phrase “excluding COVID-19”. See P.4, 66-67. |
| 4. Study Design & Participants p.5 103 Please specifiy who is meant with key stakeholders (e.g. religious/community/ etc. leaders?) | We have clarified the term stakeholders, and now it reads: “key stakeholders (e.g, policy makers, community members, and implementing partners)”. See P.6, 122-23. |
| 5. Study Design & Participants p.5 112 Incorrect spelling: take out “A” at the beginning of the sentence | We have revised the error. See P.6, 116. |
| 6. Interview process p. 6 116 incorrect spelling: degrees -> plural | Corrected. See P.7, 137. |
| 7. Interview process p.6 116: Has there been adequate training/ assistance concerning the cultural background of the study setting? | Yes. We have added information regarding the training. The one-day training was more than just focusing on interviewing techniques. The text in the manuscript now reads:  “*The research assistants received one day of training on qualitative interview techniques, the consenting process, and the contextual factors that interviewers need to consider to ensure privacy and cultural appropriateness while greeting and talking to persons with TB*.” See page P.7, lines 137-43. |
| 8. Data Analysis p.6 Font size differs? | We have revised any font size inconsistencies throughout the manuscript. |
| 9. Data Analysis p.6-7 elaborate on pseudonymization or anonymization? Elaborate on whether transcripts etc. were securely stored when transcripts and notes will be deleted etc. | We have provided more details on the pseudonymization and anonymization processes, and data security measures. See P.8&9, lines 165-170. The manuscript now reads:  “*Sequential numbers and the category of respondents were used to save transcripts and audio recordings. To protect the identity of respondents, the names of participants, institutions, and individuals mentioned during the interviews have not been reported. Research interviews and transcripts were digitally stored securely on a password-protected network drive to protect the confidentiality and privacy of participants*”.  Research interviews and transcripts will be deleted after ten years according to the data retention policies specified in the research protocol. |
| 10. Ethical considerations p.7 153 informal spelling: can’t -> cannot | Informal spelling corrected. See P.9, line 182. |
| 11. Characteristics of participants p.8 165 incorrect phrasing: include: The majority OF healthcare workers | We have revised the phrasing. See P.10, line 193. |
| 12. Facilitators of DATs uptake p.11 199 What does XX stand for at the end of the sentence? | This was added by mistake. We removed the "XX". See P.11, line 223. |
| 13. Facilitators of DATs uptake p.13 Formatting is off | Formatting issues corrected. |
| 14. Facilitators of DATs uptake p. 15 276 font and font size differs | We have revised the font and font size differences. |
| 15. Reduce workload and work-related TB exposure to providers p.16 294: missing letter: ReduceD 295 missing word add “a” few | Missing letters corrected. See P.16, 318. |
| 16. Theme 2 p.16 306 Heading is misspelled is it supposed to mean Easy for use? | Corrected the heading as "Easy to use". See P.17, line 334. |
| 17. Theme 3 p.18 351 Wording: important others? Are they referring to “important significant others”? | Corrected as "important significant others". P.19, line 380 |
| 18. Theme 3 p.18 355 wording change to: “in their TB treatment” | Corrected. See P.19, line 383. |
| 19. Theme 4 p. 19 362 missing word: can increase “THE” uptake | Missed word added. See P.19, line 391. |
| 20. Overall heading stile is not coherent | We have revised the headings throughout to ensure a consistent heading style. |
| 21. Barriers for uptake of DATS p.20 390 missing word: those “WHO” did not have phones 392 missing word: to “THE” implementation 394 missing word: can result “IN THE” fear of stigma | Corrected the missing words. See P.21, lines 418-422. |
| 22. Theme “1” Network and electricity issues p.21 397 Duplicate Heading Theme one already exists maybe it is best to change it to Theme 1.2. ? 404 wrong spelling: my? Do they mean: may? 428 PWTB is always addressed singularly needs to be plural add an “s” 447 missing word: they “HAD” forgotten.. 448: missing words may consider them “TO BE” non-adherent come to “THE” health facility | Duplicate heading: we kept as was, given the previous themes were under ‘facilitators’. The emerged themes under ‘barriers’ are numbered sequentially as well.    Spelling error corrected (p.23&24, 475-479), plural usage included, and missing words added. |
| 23. Theme 2: Lack of inclusivity p. 24 459. missing word: As “A” barrier 460 odd sentence: those did not have phones? people in prison: is that not too far-fetched since this target group is not included here? | Missing word added (p.24, 488). Sentence related to regarding people in prison kept as is given this reflects direct responses from health care workers. |
| 24. Theme 3: Fear of Stigma etc. p.24 473 missing word: was | Missing word added. P.25, 502 |
| 25. Discussion p.25 495-496 phrasing is odd | Revised for clarity. P.26, 518. |
| 26. Strenghts and limitations p. 27 537 missing word “THE” capturing 538 missing letter: engaging diverse “groupS” including “DRUG SENSITIVE PEOPLE” 50 is “THAT” it adds 547: DATS font is different | Missing words and letters, and font differences were corrected. P.30, lines 575-579. |
|  |  |
| Reviewer Two |  |
| General comment  The manuscript has important piece of information and crucial to the field that gives a hope on the impact of digital technology in helping the monitoring of medication adherence to TB treatment in Ethiopia, however, access to technology and utilization of digital platform is still limited in rural part of the country where TB is highly prevalent. | We appreciate the recognition of our manuscript's importance. We revised the manuscript as per the specific comments by the reviewer. |
| Specific comments |  |
| 1. Suggested Title: good to minimize double use of key terms, remove ‘adherence’: “Facilitators and barriers to uptake of digital technologies in improving TB treatment adherence in Ethiopia: A qualitative study” | We have revised the title and now it reads: “*Facilitators and barriers to uptake of digital adherence technologies in improving TB care in Ethiopia: a qualitative study*” |
| 2. Abstract: Page 2: line 36-38: ‘Network issue’ is appeared as barrier, but it is not specific and may not be clear for readers. Do you mean network is not clear, patient network or internet connectivity? | We have rephrased it to read as “network issue (mobile phone signal) ........”. P.2, 38. |
| 3. Background: Well described except, mix of acronym and long form inconsistently eg. DOTs and DATs | We have revised the inconsistencies throughout the document. |
| 4. Good to add some literature that shows the magnitude of TB and treatment adherence level in Ethiopia | We have included the latest data in the introduction section and details about the adherence challenges. See P.4, lines 81-83. |
| 5. Methods: As this study is nested from cluster RCT study, did this qualitative study consider both the intervention and comparison group perspectives about the technology use to track TB treatment? | This qualitative study focuses on experiences/perspectives from people with direct use of a DAT and therefore included intervention arms only. |
| 6. The reason for the purposively focusing ‘Urban setting’ is not clear at all. Although TB is common every part of the country, but disproportionately higher in Rural areas and adherence to treatment is also affected by several barriers in Rural setting. So why this study only targets Urban dwellers. Authors should give tips about this issue. | This qualitative substudy was embedded within a cluster randomised trial of health facilities conducted in two regions (Addis Ababa and Oromia). Health facilities were predominantly in urban areas; overall of the 78 health facilities in the trial, only 5 were classified as being in a rural area. This primarily was due to the selection of health facilities with at least 50 TB notifications per year to achieve our target sample size over 12-month enrolment period. This therefore resulted in health facilities being larger clinics in urban areas.  We have now added more detail in the manuscript and the number of facilities from which PWTB and health care providers were enrolled (see Annex 1 in the supplemental file). For logistical reasons, the facilities purposively selected were all from urban areas. We have added a comment in the discussion on this issue (See P.29, 581). |
| 7. Categories of study participants: Healthcare workers are also key stakeholders. Kindly check this one as well. | We have described the term “stakeholders” to add more clarity. In this manuscript, we consider “stakeholders” as policymakers, implementing partners and community advisory board members. See p.6,121. |
| 8. Page 6: line 114-121: How was the interview conducted? Where was the interview held, at Health facility or home? | We have now included more information on this. See p.7: 142-133. |
| 9. Results: There should be a section to separate the methods and results | Revised. See p.9, 183 |
| 10. Good to move the description of ‘Themes’ to Methods section | This section was kept as it was, given the “themes” emerged constitutes the results section. |
| 11. Improved patients-provider relationship through person-centred care… suggestion “Improved patients-provider relationship” | We prefer to keep this as is. No change has been made. The phrasing “person-centred care” is relevant in this context. The improved relationship between patients and providers was achieved through DATs which enabled differentiated care based on the needs and circumstances of individual patients. |
| 12. Page 16: Split the sub-topic: as ‘Reduce Healthcare providers workload’ ‘Reduce Health care providers work-related TB exposure’ | We have revised this as suggested by the Reviewer. See page p.16, lines 318-328. |
| 13. Good to have brief summary about the ‘implication of the study’ that might be good for high TB burden countries and being challenged by non- adherence issue. | Thank you for this suggestion. We have added an “implications” section under the discussion and summarised the implications of this study for practice and research. See page 28, lines 564-570. |
| Reviewer Three |  |
| I would like to commend the authors on carrying out this study which I believe will contribute to the body of knowledge in Tuberculosis treatment. The study also highlights innovative approaches to ensuring adherence in patients. I however have the following recommendations. | Thank you for the positive feedback and encouragement. We're glad our study on innovative approaches to TB treatment adherence is seen as a valuable contribution to the field. Below, we have addressed the specific comments from the reviewer. |
| 1. Introduction: Authors should ensure they discuss to some extent what the DOT is. There appears to be no attempt at educating the reader about DOT. | We have revised the introduction to include a concise explanation of the Directly Observed Therapy (DOT) approach, highlighting its purpose, methodology, and common challenges. See P.4, lines 71-74. |
| 2. Study setting: The study focused more on urban settings. No mention of a rural setting was made. Is Oromia rural? Was the mention of Oromia in this sentence as an urban area an error? “The study sites in Addis Ababa were all urban while predominantly urban in Oromia” | This qualitative substudy was embedded within a cluster randomised trial of health facilities conducted in two regions (Addis Ababa and Oromia). Health facilities were predominantly in urban areas; overall of the 78 health facilities in the trial, only 5 were classified as being in a rural area (all in Oromia). This primarily was due to the selection of health facilities with at least 50 TB notifications per year to achieve our target sample size over a 12-month enrolment period. This therefore resulted in health facilities being larger clinics in urban areas (see Annex 1 in the supplemental file).  For logistical reasons, the facilities purposively selected for this qualitative substudy were all from urban areas. We have added a comment in the discussion on this issue. See P.29, line 581 |
| 3. The authors should please make an attempt to describe the Everwell platform in a bid to educate the reader. | We have added a detailed description of the Everwell platform explaining its functionality and role in monitoring patients' medication adherence. See P.6, lines 105-115 |
| 4. Study design and participants: An additional group composed of relatives of persons with TB would have served as an important demographic to consider for the Interview process. | We acknowledge this valuable point. We have highlighted this limitation in our manuscript. See P.29, line 585. |
| 5. As the training for the research assistants lasted for a day, could you please share the degree of agreement between the interviewers and the trainees? 6. Did you have mock trials on more participants to see if the research assistants acquired the skills for data collection? | At the end of the one-day training, the research assistants conducted mock interviews among themselves to practice and refine their skills. Feedback was provided by the trainers, ensuring that any misunderstandings or errors were promptly addressed. Additionally, after each field interview, debriefing sessions were held where the research assistants had the opportunity to discuss their experiences, share insights, and receive further guidance. This continuous learning process allowed them to deepen their understanding and improve their application of the interview protocols and techniques. These combined efforts demonstrated that the trainees effectively grasped and could competently implement the methodologies covered during the training, resulting in a high degree of agreement between the interviewers and the trainees.  We have included a brief description in the manuscript. See P. 7, lines 137-143 |
| 7. How did you ensure inter-rater variation? | To ensure inter-rater reliability, we used double coding of a subset of interviews, followed by discussion and consensus meetings to resolve any discrepancies. This process is detailed in the methodology section. See P.8, 154-157. |
| 8. In addition, a pilot study may also have helped to guarantee that the research assistants after the training were capable of conducting the study. | As described above, interviewers have taken trainings and conducted mock trial prior to the main study to refine the interview process and ensure research assistants were adequately trained. |
| 9. Proper description of 'smart pill box' and the 'medication labels' could have been described in the methodology. | We have revised the methodology section to include a comprehensive description of the 'smart pillbox' and 'medication labels,' detailing their design, functionality, and usage in the study. See P.6, 105-115. |
| 10. I will suggest that the process of monitoring patients' use of medication be described in detail in the methodology. | We have expanded the detail on the process used to monitor patients' medication use, including the roles of the Everwell platform, text message reminders, and follow-up procedures. See P6. 105-115. |
| 11. Please share what objective method was used to confirm that the patients’ actually used their medication beyond the technological methods of sending a text message? | The TB digital adherence technologies rely on proxy measures to monitor adherence. For instance, the smart pillboxes send a signal when the box is opened, indicating that the patient has accessed their medication. Similarly, with the medication labels, patients send a code each time they take their medication, which is logged by the system to confirm adherence. While these technological methods provide valuable data, they do not directly confirm the ingestion of the medication.  We have included more details in the methods on the two DAT interventions See P6. 105-115. |
