# Supplementary material for: Facilitators and barriers to uptake of digital adherence technologies in improving TB care in Ethiopia: A qualitative study
Source: PLOS Digit Health. 2024 Nov 21;3(11):e0000667. doi: 10.1371/journal.pdig.0000667 (PMC11581308; doi:10.1371/journal.pdig.0000667)
Supplement: Attachment — Submitted filename: Response to Reviewers1.docx [file pdig.0000667.s006.docx]

To: Margaret Isioma Ojeahere, MBBS, FWACP
Academic Editor, PLOS Digital Health

24 September 2024

Dear Dr Ojeahere

Re: PDIG-D-23-00479 (Facilitators and barriers to uptake of digital adherence technologies in improving TB treatment adherence in Ethiopia: A qualitative study)

We would like to thank the reviewers for their comments, and we are honoured to be given the opportunity to respond to the points they raised. We have outlined our responses to all comments below and indicated where there are major changes in the text.

Yours sincerely

Zewdneh Shewamene on behalf of all co-authors

| Reviewer Comments | Response |
| --- | --- |
| Reviewer One |  |
| Reviewer #1: The research article has been properly adapted to the reviewers comments. I have added a few minor changes as comments in the attached pdf revision version. I would ask to please check the general submission guidelines provided by the journal (an example can be found on the official website). This concerns the font size, the headings for the figures etc. | We would like to thank the Reviewer for their thoughtful review and positive feedback. We appreciate their acknowledgment of the revisions made in response to their comments. Their previous recommendations were instrumental in refining this manuscript, and we are grateful for their valuable contributions.  The minor changes suggested in the attached PDF have been carefully reviewed and addressed (we extracted the comments from the pdf as shown below).  We have ensured compliance with the journal's submission guidelines, including for font size and figure titles. |
| 1. Page 4, line 67. I would rephrase, „became the primary cause“ cause of what? | Revised as “…primary cause of death.” (See page 4, line 67). |
| 2. Page 4, line 74. missing space between process and daily | Revised. Space added between the words (see page 4, line 73). |
| 3. Page 7, line 142. maybe say „practice interviews“ or „preliminary interviews“ instead | Revised to practice interviews. See page 7, line 142. |
| 4. Page 19, line 390. capital letter | Revised. “p” for policy has been capitalized (see page 19, line 390). |
| 5. Page 24, line 490. if it is taken from an interview statement, could you include the statement in here? | An additional direct quote was provided to support the statement (see page 25, lines 501-503). |
| 6. Page 28, line 570-71. could you elaborate on what you mean with „several opportunites and initiatives“? Could you also include implications for research next to pracitse? | The statement is now elaborated and reads as “*Despite these challenges, several opportunities and initiatives, including the presence of TB care coordination platforms at national and regional levels, health digitization initiatives by the MOH, and the MOH's commitment to funding new TB programs, could support the feasible implementation, adaptation, and scale-up of DATs in the Ethiopian context*.” (See page 28, lines 573-76).  Implication for research is added (See page 28, lines 576-79). |
| 7. Page 29, line 597. again what exactly would those be? | The sentence is revised and read “Despite those barriers, key policy stakeholders believe there are several opportunities and initiatives that could support the feasible implementation, adaptation, and scale-up of DATs in the Ethiopian context, including the MOH's commitment to expanding health digitization efforts and funding new TB programs” (See page 30, lines 604-607). |
| Reviewer Three |  |
| Reviewer #3: A significant amount of the recommendations have been attended to. | We thank the Reviewer for their valuable feedback and confirming their recommendations have been addressed. Their insights have been instrumental in refining our work. |
| Reviewer 4 |  |
| Reviewer #4:  1. page 5, line 90-92. sustainability should be changed to "sustainably"  2. page 30, line 600-601. Punctuation to be done properly by inserting comma. "Oromia regional, state....). Woreda health officer changed to "ward health officers" | We sincerely thank the Reviewer for their valuable feedback and insightful comments, which have greatly contributed to improving the quality of our manuscript.  Revised (see Page 5, line 92).  Rephrased the wordings and punctuation revised (See page 30, line 611-12). ‘Woreda health office’. ‘Woreda” is an administrative division of Ethiopia |
